# Supplementary material for: Multiple Porcine Innate Immune Signaling Pathways Are Involved in the Anti-PEDV Response
Source: Viruses. 2023 Jul 26;15(8):1629. doi: 10.3390/v15081629 (PMC10458394; doi:10.3390/v15081629)
Supplement: Supplementary file 1 [file viruses-15-01629-s001.zip › viruses-2517746-supplementary.pdf]

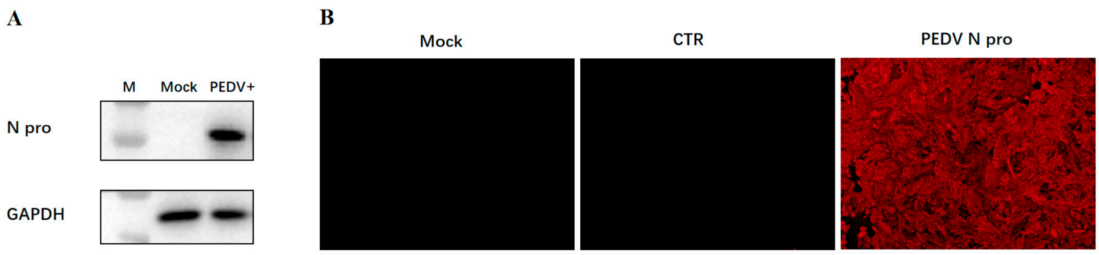

Figure S1. The specific recognition of PEDV in Vero cells by PEDV N mAb in WB detection (A) and in IFA detection (B). Mock was cells uninfected with PEDV, and CTR was cells infected with PEDV but not incubated with N protein mAb as the negative control.

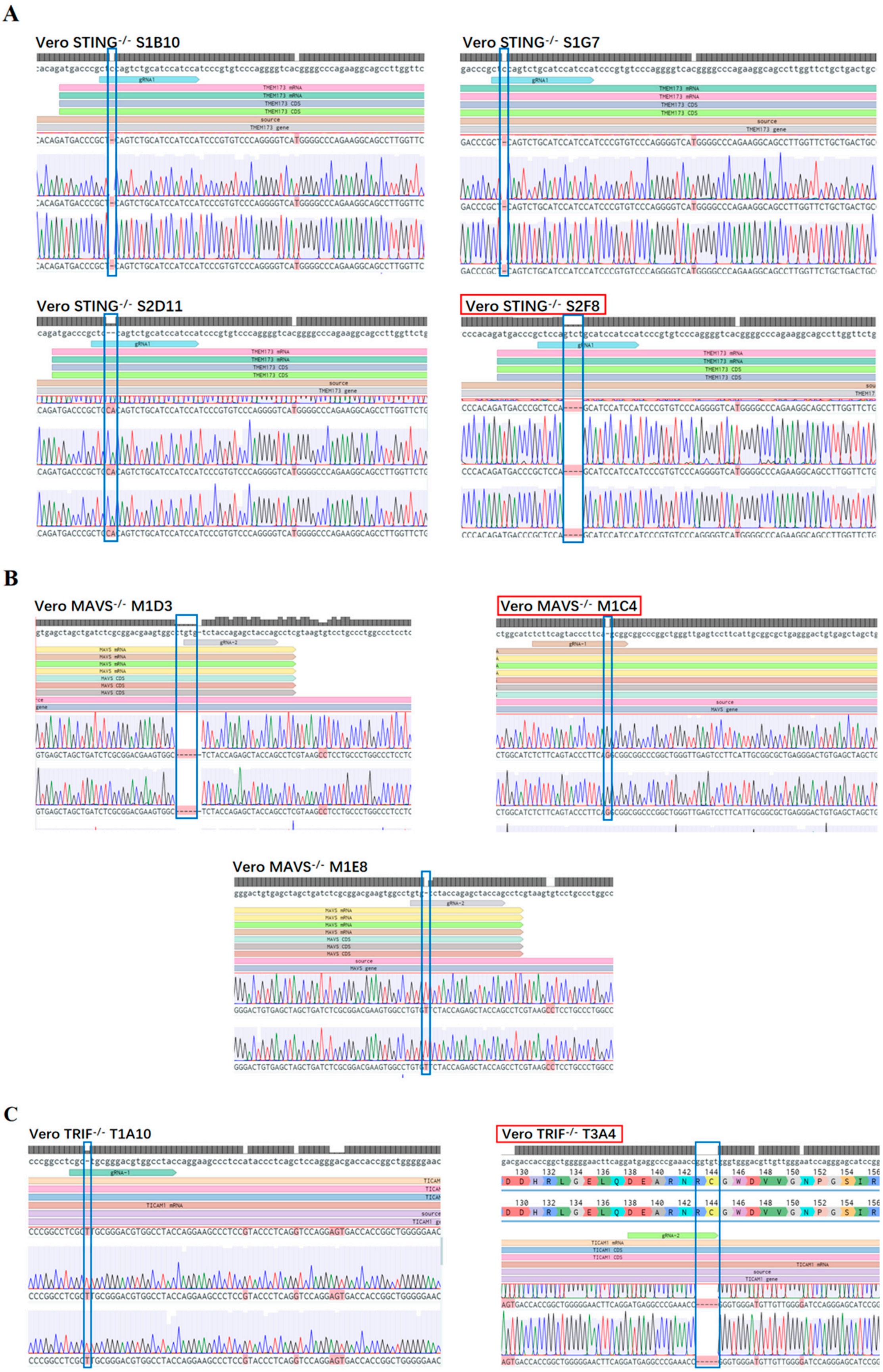

Figure S2. The genome sequencing results and characterization of STING, MAVS and TRIF KO cell clones. The STING (A), TRIF (B), and MAVS (C) PCR products of individual KO cell clones were

cloned into T vectors and the multiple cloned sequences were aligned with the corresponding template genomic DNA sequences for analysis of base insertion/deletion (ins/del). For each cell clone, two typical sequences are shown for alignment, with the ins (+) and del (-) are blue boxed. A, the clones S1B10, S1G7, S2D11 and S2F8 are all STING$^{-/-}$ cells. B, the clones M1D3, M1C4 and M1E8 are all MAVS$^{-/-}$ cells. C, both T1A10 and T3A4 clones are TRIF$^{-/-}$ cells. The red boxed KO cell clones were selected for formal experiments.

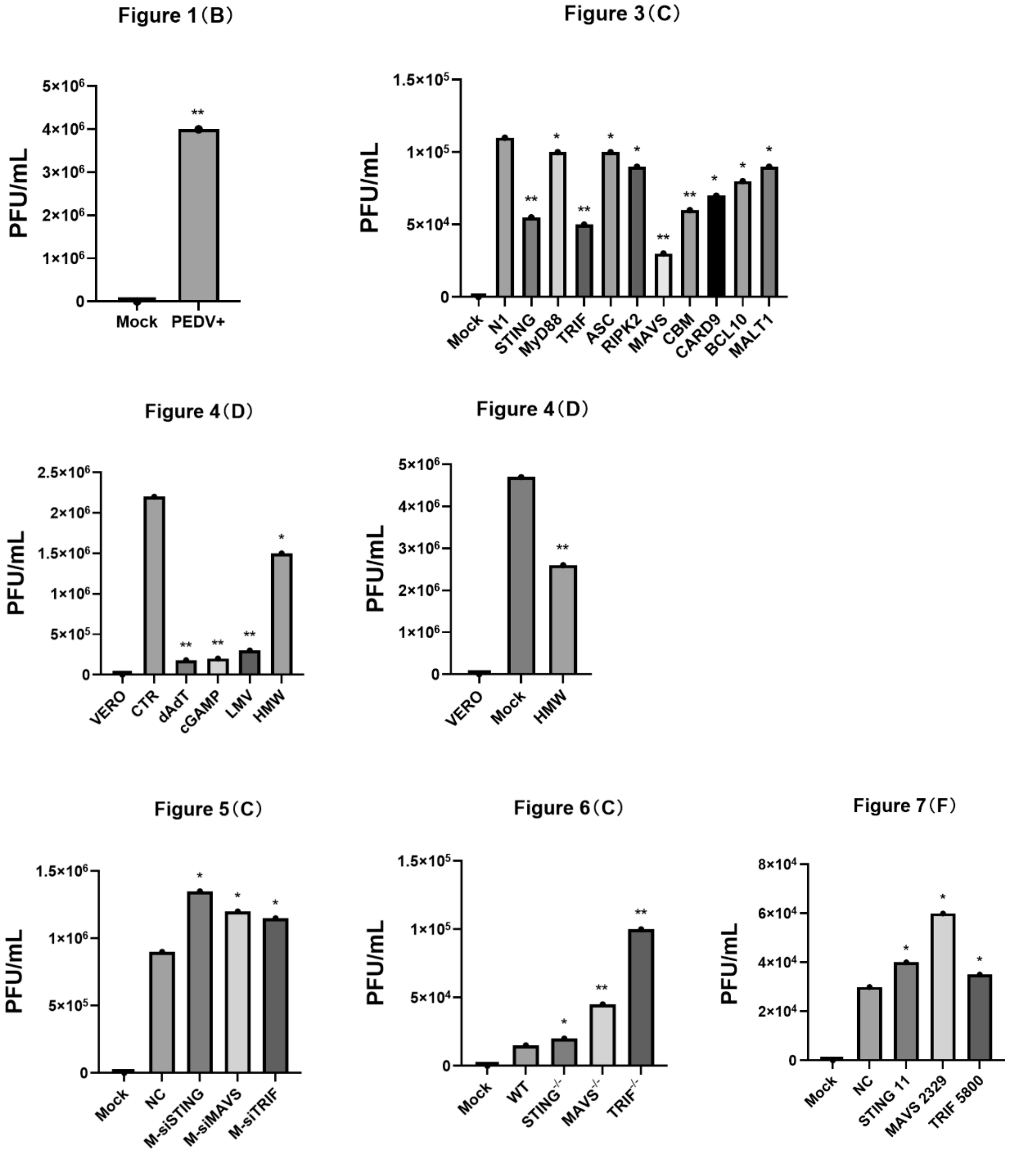

Figure S3. The quantitative column diagrams of the plaque assays in this study. All the results of the plaque assays were from repeated experiments, with Fig 3C more than 3 repeated experiments and the rest 2-3 repeated experiments. The $p < 0.05$ (*) and $p < 0.01$ (**) versus Mock or CTR in Fig 1B, Fig 3C and Fig 4D; versus NC or WT Fig 5C, Fig 6C and Fig 7F.
